# Marine Polyphenol Phlorotannins as a Natural Sleep Aid for Treatment of Insomnia: A Review of Sedative–Hypnotic Effects and Mechanism of Action

**DOI:** 10.3390/md20120774

**Published:** 2022-12-10

**Authors:** Seonghui Kim, Duhyeon Kim, Min Young Um, Minseok Yoon, Jae-Suk Choi, Yung Hyun Choi, Suengmok Cho

**Affiliations:** 1 Department of Food Science and Technology, Institute of Food Science, Pukyong National University, Busan 48513, Republic of Korea; 2 Research Division of Food Functionality, Korea Food Research Institute, Wanju 55365, Republic of Korea; 3 Department of Seafood Science and Technology, The Institute of Marine Industry, Gyeongsang National University, 38 Cheondaegukchi-gil, Tongyeong-si 53064, Republic of Korea; 4 Department of Biochemistry, College of Korean Medicine, Dong-Eui University, Busan 47227, Republic of Korea; 5 Anti-Aging Research Center and Core-Facility Center for Tissue Regeneration, Dong-Eui University, Busan 47340, Republic of Korea

**Keywords:** phlorotannin, marine polyphenol, sleep, insomnia, GABAergic mechanism

## Abstract

Insomnia is a common sleep disorder. Natural sleep aids are gaining worldwide popularity as alternatives to prescription drugs for improving sleep. Recently, numerous studies have investigated the sedative–hypnotic effects of the polyphenols of terrestrial plants. The hypnotic effects of marine polyphenols have also been studied in recent years. Phlorotannins are marine polyphenols that are found only in brown algae. Phlorotannins exert sedative–hypnotic effects via the gamma-aminobutyric acid type A-benzodiazepine receptor. In addition, the brown seaweed *Ecklonia cava* supplement containing phlorotannins has been approved by the Ministry of Food and Drug Safety as a health-functional ingredient that helps improve sleep quality. Currently, it is meaningful to deal with the sedative–hypnotic effects of phlorotannins as natural sleep aids. The current review comprehensively presents the sedative–hypnotic effects in animal models and human clinical trials as well as their mechanism of action, extraction, purification, and safety.

## 1. Introduction

Polyphenols are one of the most common classes of secondary metabolites found in terrestrial and marine plants [1]. Polyphenols from terrestrial plants and marine algae have different chemical structures [2]. Phlorotannins are a major polyphenolic class found only in brown algae, whereas red and green algae contain the most phenolic compounds, such as flavonoids, phenolic acids, and bromophenols [3]. Phlorotannins are oligomers and polymers of the monomeric unit phloroglucinol (1,3,5-tri-hydroxybenzene) with molecular weights in the range of 250–1738 Da [4,5]. They are an extremely diverse group, and individual phlorotannin compounds are structurally similar [5]. To date, approximately 150 phlorotannins have been isolated from various brown seaweeds [6,7].

In the last three decades, phlorotannins have been extensively investigated and shown to possess various biological properties including antioxidative, antidiabetic, anti-aging, anti-inflammatory, anti-allergic, neuroprotective, and memory-enhancing properties [7,8,9,10,11,12,13,14,15,16]. However, the sedative–hypnotic effects of phlorotannins have only recently been studied [4,17,18,19]. It has been demonstrated that phlorotannins from brown seaweeds have hypnotic effects in in vitro and in vivo studies as well as in clinical trials. Studies on the hypnotic effects of phlorotannins have shown their characteristics as agonists for gamma-aminobutyric acid type A (GABA_A_)-benzodiazepine (BZD) receptors. Several researchers have reviewed the biological properties of marine polyphenol phlorotannins; however, their hypnotic effects have not been reviewed.

Sleep deprivation and disorders, such as insomnia, are now associated with numerous serious health problems and are appraised as emerging global epidemics that cause social and financial burdens [20]. As insomnia becomes more common, herbal sleep aids are gaining popularity worldwide as alternatives to prescription drugs to treat insomnia or improve sleep quality [21,22]. Most sedative–hypnotic drugs have numerous side effects, such as impairment of memory, cognitive function, and general daytime performance; therefore, their use is generally not recommended beyond 4 weeks [23,24]. In addition, long-term administration typically results in dependence and tolerance [25]. Thus, sedative–hypnotic effects of herbal plants or their phytochemicals have been widely reported, such as valerian (*Valeriana officinalis*), St. John’s wort (*Hypericum perforatum*), kava kava (*Piper methysticum*), passion flower (*Passiflora incarnata*), and hops (*Humulus lupulus*) [19,24]. Although numerous studies have been conducted on the hypnotic effects of herbal plants, few studies have investigated marine polyphenol phlorotannins. Currently, it is noteworthy to mention the sedative–hypnotic effects of the marine polyphenol phlorotannins. This review aims to present the extraction, purification, safety, sedative–hypnotic effects, and mechanism of action of phlorotannins.

## 2. Extraction and Purification of Phlorotannins

Phlorotannins from brown seaweeds have been extracted using traditional extraction techniques (Soxhlet, solid–liquid, and liquid–liquid extractions), enzymatic hydrolysis, and solvent extraction [26,27]. Currently, the solvents used in extraction methods should be non-toxic and inexpensive [28]. Ethanol extraction is the preferred method in the food industry because of its safety for human consumption, the convenience of processing, and low cost [29]. The correct selection of the extraction solvent, solvent concentration, temperature, and time are variables that directly influence the yield of biologically active compounds. To determine the scale-up, it is important to alter different parameters to optimize the extraction process [30]. One of the most consistent multivariate techniques in analytical optimization is response surface methodology [31]. According to a report by Yoon et al. [26], in which the sedative–hypnotic compound was extracted from *Ecklonia cava*, the active total phlorotannin content, yield of phlorotannins, and sleep duration were independent variables [26]. Sleep duration and total phlorotannin content were highly correlated (*R*^2^ = 0.9102), and the optimal conditions for extraction time, extraction temperature, and ethanol concentration were 22.8 h, 80 °C, and 95.0%, respectively [26]. The optimal conditions for the yield of phlorotannins were 24.0 h extraction time, 80 °C, and 88.3% humidity. There were several differences between the hypnotic effect under optimal conditions and the sleep effect under other conditions. Following optimization, the total phlorotannins and yield of phlorotannins were approximately 570 mg phloroglucinol equivalents per gram (mg PGE/g) and 7.5%, respectively, which were 1.8- and 1.5-fold higher than the 315.4 mg PGE/g and 4.9% obtained under the conditions that showed the lowest results.

Brown algae products are considered a major safety concern for arsenic [32]. In particular, brown algae have the highest arsenic concentration, whereas red and green algae have phlorotannin-chelating activity [33]. According to the Ministry of Food and Drug Safety (MFDS), the acceptable daily intake (ADI) of arsenic is 150 μg for a person weighing 60 kg. High arsenic intake can cause numerous health concerns, including skin and lung cancers [34], hyperkeratosis [35], diabetes [36,37], and vascular diseases [38]. In 2014, Kim et al. [39] reported that 1 g of crude phlorotannin extract included 180 μg arsenic. These results show that, according to the MFDS, the crude phlorotannin extract exceeded the ADI of arsenic. Several efforts have been made to reduce arsenic through purification [40]. Macroporous adsorption resins have been extensively used to purify phytochemicals and bioactive compounds from food and plant extracts due to their high adsorption capacity, easy recyclability, and various functional groups [41]. These resins can be used for the absorption of organic constituents because of their weak polar and hydrophobic properties [42]. A previous study showed that the arsenic content of the final phlorotannin product was 48 μg/g, which was 3.75-fold lower than that of the crude phlorotannin extract purified using HP-20 resin [39]. These results suggest that the purification of phlorotannins using HP-20 resin is effective for arsenic removal.

## 3. Safety and Toxicity of Phlorotannins

### 3.1. In Vitro

In human and animal cell lines, such as human epidermal (HaCaT), Henrietta Lacks (HeLa), human colon adenocarcinoma (Caco-2), highly tumorigenic (HT1080), HepG2, B16F10 melanoma, KU812, RBL-2H3, MRC-5, HT-29, human fibroblast cells, and rat vibrissae immortalized dermal papilla cell line [43,44,45,46,47,48,49,50,51,52,53,54,55,56,57], phlorotannins decreased the generation of reactive oxygen species (ROS), malondialdehyde levels, deoxyribonucleic acid (DNA) damage, and ultraviolet B (UVB) radiation-induced damage. In addition to these activities, phlorotannins also reduce binding between immunoglobulin E (IgE) and the high-affinity IgE receptor as well as the expression of several genes, including tumor necrosis factor alpha, interleukin-1β (IL-1β), IL-6, IL-8β expression, prostaglandin E2 (PGE2) release, cyclooxygenase 1 (COX-1), COX-2, microsomal prostaglandin E synthase-1 (mPGES-1), nuclear factor-kappa B, activator protein-1 reporter, the mitogen-activated protein kinase (MAPK) signaling pathway, and melanin synthesis. Moreover, phlorotannins have been reported to inhibit the growth of HeLa, A549, HT1080, and HT29 tumor cells. Phlorotannins also inhibit 5-reductase activity and increase cell viability and glutathione concentration. Notably, to the best of our knowledge, studies have reported that phlorotannins exhibit biological activities without toxicity in human and animal cell lines.

### 3.2. In Vivo

In animals, the safety and toxicity of phlorotannins have been evaluated in fish, such as seabream (*Pagrus major*), tiger puffer (*Fugu rubripes*) [58], zebrafish (*Danio rerio*) embryos [59], and zebrafish [60]; in rodents, such as Institute of Cancer Research (ICR) mice [60,61], HR-1 hairless male mice [46], and Sprague–Dawley (SD) rats [61,62]; and in Beagle dogs [63]. In fish, phlorotannins reduced ROS levels, cell death, generation of thiobarbituric acid reactive substances, and adipogenic factors, such as peroxisome proliferator-activated receptors (PPAR), CCAAT-enhancer-binding proteins (C/EBP), fatty acid-binding protein 11a (FABP11a), and sterol regulatory element-binding factor-1 (SREBF-1) with minor side effects including writhing and gasping for several seconds (after which the fish calmed down) and some discharged oral mucus. However, the survival rate of these fish was 100% [58,59,60]. In rodents, phlorotannins reduce the final body weight, the high-fat diet-induced elevation of liver fat, low-density lipoprotein cholesterol [60], lipid peroxidation, protein carbonylation, epidermal height, and MAPK expression [46]. Phlorotannins also increased the levels of plasma triglycerides, total cholesterol [60], and α-amylase to the normal range [62] and increased the survival rate of rodents until the end of the experiments. In Beagles, mild side effects such as soft stool and diarrhea were reported after phlorotannin treatment. However, the survival rate of the Beagles was 100% at the end of the treatment [63]. Further research is needed to confirm the potential for phlorotannins as health-functional feed agents and in veterinary medicine for various animal species.

### 3.3. Clinical Human Studies

In humans, phlorotannins can be used as food supplements and functional food ingredients. Phlorotannins have been reported to possess numerous advantages [64,65,66] and mild side effects [64]. A study by Paradis et al. [66] found that phlorotannins isolated from *Fucus vesiculosus* and *Ascophyllum nodosum* decreased the incremental areas under the curve for plasma insulin, the post-load plasma insulin concentration, the plasma glucose area under the curve, and the postprandial insulin concentration in 23 participants following treatment with 250 mg/capsule. Moreover, phlorotannins elevated the level of a surrogate marker for insulin sensitivity in all participants.

A study by Baldrick et al. [65] reported that phlorotannins extracted from *A. nodosum* decreased DNA damage and did not significantly improve C-reactive protein, antioxidant status, or inflammatory cytokines in 80 participants between 30 and 65 years old following administration of 100 mg/capsule for 8 weeks. Similarly, Shin et al. [64] reported that phlorotannins isolated from *E. cava* decreased the total cholesterol/high-density lipoprotein cholesterol level, body fat ratio, atherogenic index, total cholesterol/low-density lipoprotein cholesterol level, body mass index, waist circumference, and waist/hip ratio in 107 participants (138 men and 69 women) following administration of 72 and 144 mg/capsule. In another study, phlorotannins successfully increased sleep duration scores and inhibited the onset of wakefulness after sleep [67]. However, phlorotannins showed no serious adverse effects, such as mild fatigue, dizziness, nausea, and abdominal distension [67]. The mechanisms of action of other classes of phlorotannins that have not been tested should be further investigated to evaluate their potential as novel pharmaceutical agents for humans.

### 3.4. The Regulation of Phlorotannins as Human Supplements

The European Food Safety Authority Panel on Dietetic Products, Nutrition, and Allergies, pursuant to Regulation No. 258/97, announced that novel food supplements from phlorotannins (marketed as SeaPolynolTM) are safe for human consumption [68]. The application of phlorotannins as food supplements and functional food ingredients was reported by Turck et al. [68] and Catarino et al. [69]. As a food supplement, the daily intake of phlorotannins depends on the age of the consumer. In adolescents (12–14 years of age), the maximum daily intake was 163 mg/day. For those above 14 years of age and adults, the daily intakes were 230 mg/day and 263 mg/day, respectively.

*E. cava* extract is the main ingredient of Seanol-F sold by Simply Healthy LLC. (Leander, TX, USA) and was reported as a New Dietary Ingredient by the US Food and Drug Administration (FDA) in 2008 [70]. Daily intake was 47 mg/day for those aged >12 years. In 2015, the MFDS recognized *E. cava* extract (No. 2015-6) as a functional ingredient in health-functional foods that helps improve sleep quality [71].

## 4. Sedative–Hypnotic Effects of Phlorotannins in Animal Models

### 4.1. Phlorotannin Preparations

Various phlorotannin preparations, including ethanol and enzymatic extracts, and purified phlorotannin supplements have been investigated to evaluate their sedative–hypnotic effects [17,72,73]. Additionally, several solvent fractions from *E. cava* ethanol extracts have been shown to have hypnotic effects [17]. Ethanol [17] and enzymatic [72] extracts decreased sleep latency and increased sleep duration, respectively, in a pentobarbital-induced sleep test in mice. In a study by Cho et al. [73], phlorotannin supplementation with 90% phlorotannin potentiated sleep induced by pentobarbital in a dose-dependent manner. Among the solvent fractions (hexane, ethyl acetate, and butanol), the ethyl acetate fraction, which was characterized as a polyphenol-rich fraction, showed the best hypnotic effect. These results indicate that phlorotannins are responsible for the sedative–hypnotic effects of brown seaweed extracts or phlorotannin supplementation.

**Figure 1:**
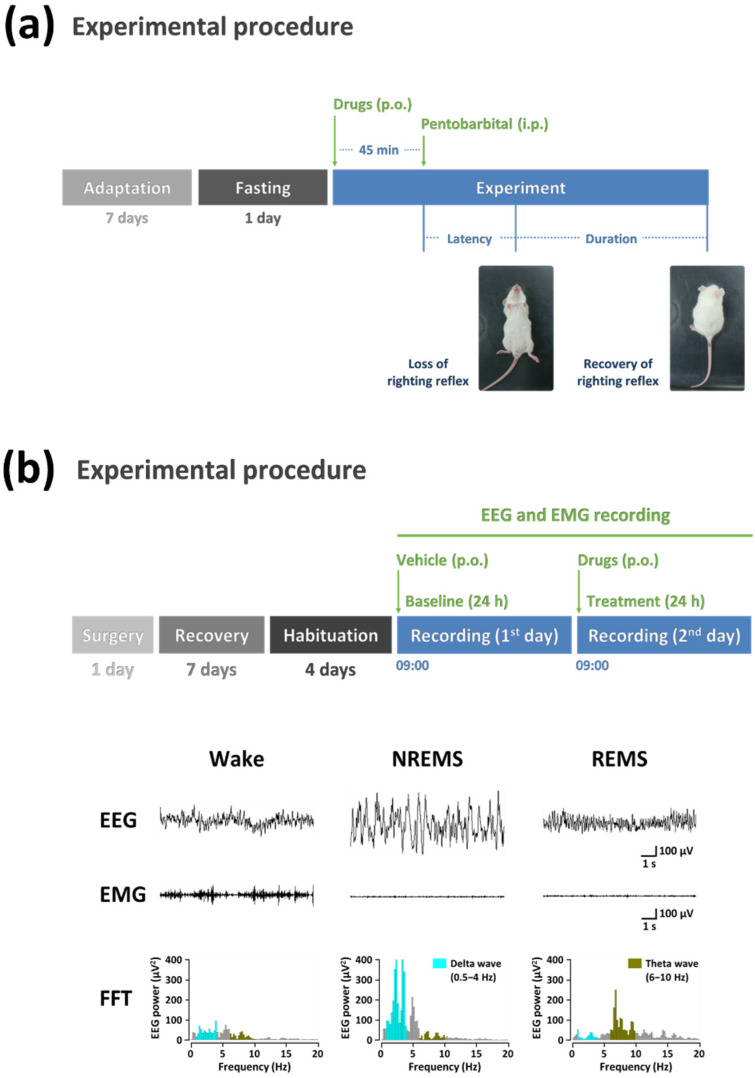
Schematic illustrations of (**a**) pentobarbital-induced sleep test and (**b**) polygraphic recordings. Abbreviations: p.o., post-oral injection; i.p., intraperitoneal injection; EEG, electroencephalogram; EMG, electromyogram; FFT, fast Fourier transform.

The pentobarbital-induced sleep test is a well-known method to assess suspected sedative–hypnotic activity [74,75] (Figure 1). However, it is difficult to identify only pentobarbital-induced sleep tests because the hypnotic effects of compounds can be induced by toxicity or other side effects [76]. In addition, this method only evaluates sleep quantity, such as sleep latency and sleep duration. Meanwhile, an analysis of sleep structure based on polygraphic recordings can verify sleep quality, including delta activity during non-rapid eye movement sleep (NREMS), and sleep–wake profiles [77] (Figure 1) (Table 1).

In the polygraphic recordings, *E. cava* ethanol extract at 500 mg/kg significantly increased the amount of NREMS by 71.4% during the first 2 h after oral administration [78]. In addition, phlorotannin supplementation at doses of 250 and 500 mg/kg significantly increased the amount of NREMS 2 h immediately after oral administration [4]. The phlorotannin supplement (500 mg/kg) showed sleep-promoting effects similar to those of diazepam (6 mg/kg). However, while diazepam reduced the electroencephalogram (EEG) power density of NREMS (frequency range, 0.5–4 Hz), phlorotannin supplementation did not show any significant difference. These results suggest that phlorotannin supplementation induces natural sleep without adverse effects following the onset of sleep [79].

Sedative–hypnotic effects can be evaluated using a caffeine-induced sleep disruption model [8]. Caffeine promotes wakefulness by blocking the activation of the adenosine A_2A_ receptor [80,81]. Oral administration (500 mg/kg) of the phlorotannin supplement attenuated caffeine (25 mg/kg)-induced sleep disruption, and its effects were comparable to those of the hypnotic drug zolpidem (10 mg/kg). This result implies that phlorotannin supplementation may be useful in relieving the transitory insomnia symptoms caused by caffeine consumption.

### 4.2. Individual Phlorotannin Compounds

**Figure 2:**
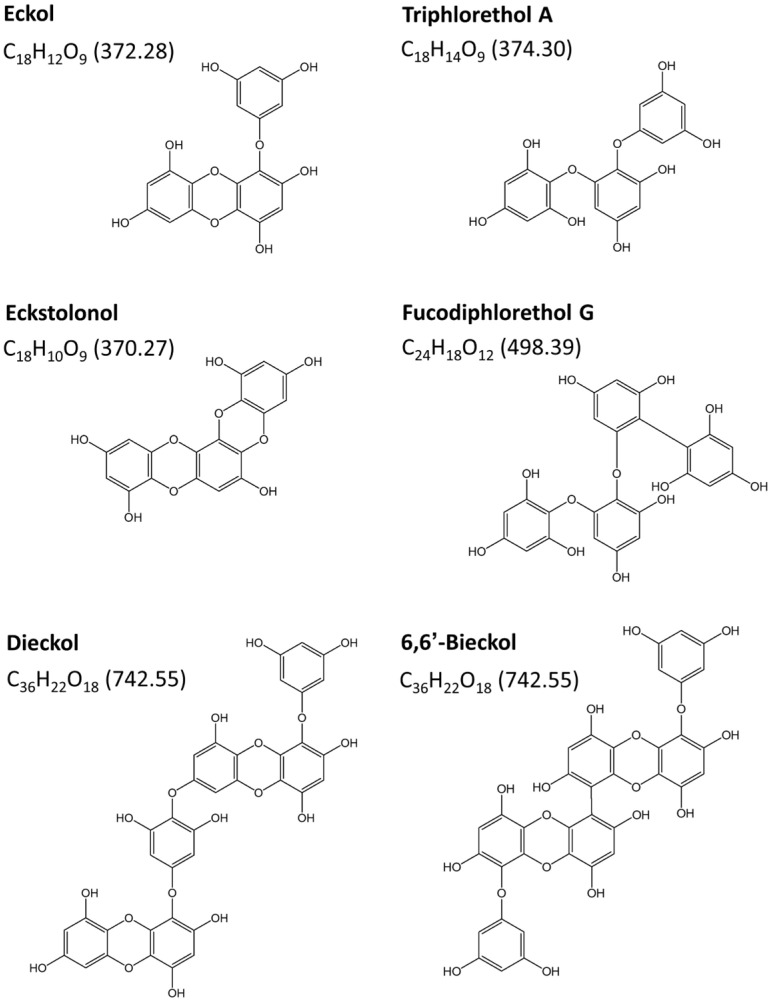
Molecular structure and molecular weight of the individual constituents of phlorotannins.

Phlorotannins are an extremely heterogeneous group, and approximately 150 different phlorotannin compounds have been isolated from various brown seaweeds [6,7,82]. Among the phlorotannin constituents, the six major phlorotannins (dieckol, eckstolonol, eckol, triphlorethol A, fucodiphlorethol G, and 6,6′-bieckol) were found to have sedative-hypnotics (Figure 2) [4]. All six phlorotannin compounds (50 mg/kg) significantly increased the sleep duration in mice treated with a hypnotic dose of pentobarbital (Table 2).

In particular, dieckol is the most abundant phlorotannin preparation from brown seaweeds and has been considered an indicator compound [39]. Yoon et al. [19] reported that dieckol has sleep-enhancing effects by analyzing its effects on the sleep–wake profiles of C57BL/6N mice using the recorded EEG and electromyogram (EMG). Dieckol administration increased NREMS duration dose-dependently. Dieckol (100 and 150 mg/kg) significantly increased NREMS levels 2 h after administration. In particular, there were no significant differences in NREMS or sleep latency between dieckol (150 mg/kg) and zolpidem (10 mg/kg). In addition, there were no significant differences in EEG power density (0–20 Hz) and delta activity (frequency range of 0.5–4 Hz) of NREMS between dieckol and the vehicle, whereas zolpidem decreased delta activity. These results imply that dieckol increases sleep quantity without inducing any adverse effects. Eckstolonol and triphlorethol A were also analyzed for their effects on sleep–wake profiles [4,83]. Eckstolonol (50 mg/kg) and triphlorethol A (50 mg/kg) significantly decreased sleep latency and increased the amount of NREMS in C57BL/6N mice, without affecting delta activity (0.5–4 Hz), similar to dieckol. Eckstolonol induced sleep effects via a GABAergic mechanism; however, the inducing effects in NREMS were moderate compared to diazepam (6 mg/kg). Triphlorethol A (50 mg/kg) showed no significant difference from zolpidem (10 mg/kg) in NREMS (Table 2).

Phlorotannins are oligomers and polymers of phloroglucinol (1,3,5-tri-hydroxybenzene), and approximately 150 phlorotannins have been isolated from various brown seaweeds. However, among the individual phlorotannin compounds, in vitro and in vivo studies have only been conducted on the six major phlorotannins (dieckol, eckstolonol, eckol, triphlorethol A, fucodiphlorethol G, and 6,6′-bieckol). Therefore, it is necessary to investigate the hypnotic effects of phloroglucinol, which is the basic structural unit of a phlorotannin, and to study further the synergistic effects of phlorotannin compounds.

## 5. Sleep-Promoting Effects of Phlorotannins in Clinical Trials

The promising sleep-promoting effects of phlorotannins have also been observed in humans. A clinical case study demonstrated the effects of acupuncture therapy and the phlorotannin-rich *E. cava* extract (500 mg/day) on sleep disturbance in patients with amyotrophic lateral sclerosis (ALS) [84]. After 5 months of combined treatment, the Pittsburgh Sleep Quality Index (PSQI) score decreased from 13 to 8 in patients with ALS. Additionally, in a randomized, double-blind, placebo-controlled trial, the effectiveness and safety of phlorotannins at a dose of 500 mg/day for 7 d in adults with self-reported sleep disturbances were investigated [67]. Sleep parameters were assessed at baseline and 1 week using the PSQI and polysomnography (PSG). Um et al. reported that phlorotannin supplementation significantly increased the “Sleep duration” scores compared to those in the placebo group. However, there were no significant differences in total PSQI scores. PSG recordings revealed that wakefulness after sleep onset, total wake time, and the respiratory disturbance index during supine rapid eye movement sleep (REMS) were significantly lower in the phlorotannin group than in the placebo group. There were no serious adverse effects, and some side effects did not correlate with the consumption of phlorotannins. However, because this sample size was small and the treatment period was limited to 7 days, a large-scale controlled/long-term clinical trial is required. In addition, metabolomics studies are required to explain how phlorotannins affect sleep after intake in the human brain. Finally, this evidence provides insights into the physiological function of phlorotannins, suggesting that they might be used as a natural sleep agent.

## 6. Action Mechanism of Phlorotannins

**Figure 3:**
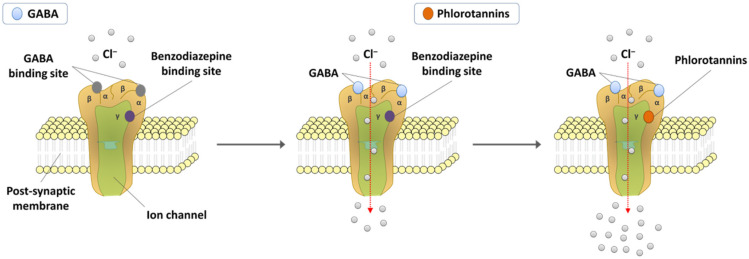
Sleep-inducing mechanism of phlorotannins.

Previous in vitro and in vivo studies have demonstrated that both preparations and the individual constituents of phlorotannins exert sedative–hypnotic effects via a GABAergic (gamma-aminobutyric acid-mediated) mechanism (Figure 3). The BZD-binding site of the GABA_A_ receptor has been considered the most important molecular target for the development of sedative–hypnotic drugs [85,86]. Currently, BZD agonists are the most commonly prescribed hypnotics. These agents act as positive allosteric modulators as BZD ligands; potentiate GABA-mediated inhibitory neurotransmission, which results in membrane hyperpolarization by allowing chloride anion (Cl-) influx; and, subsequently, exhibit sedative–hypnotic effects [87,88]. Similar to BZD agonists, phlorotannins induce sleep by acting as allosteric agonists of GABA_A_ receptors and binding to the BZD-binding site of GABA_A_ receptors.

The in vitro GABAergic mechanism of phlorotannins was demonstrated by the ligand-binding and functional assays of GABA_A_ receptors. In the GABA_A_-BZD receptor-binding assay, ethanol, methanol, and enzymatic extracts from the brown seaweed *E. cava* were significantly displaced [3H]-flumazenil binding [17,72]. Among the three extracts containing phlorotannins, the ethanol extract had the lowest half-maximal inhibitory concentration (IC_50_) (0.127 mg/mL) [17]. The binding affinities (IC_50_) of the ethyl acetate fraction [17] and purified phlorotannin supplement [4] from the *E. cava* ethanol extract were 0.019 and 0.012 mg/mL, respectively. In addition, four phlorotannin compounds (eckstolonol, eckol, triphlorethol-A, and dieckol) were identified as ligands for the BZD-binding site of GABA_A_ receptors [17]. The significant results of the binding assay imply that phlorotannins act as BZD ligands to GABA_A_ receptors; however, they cannot provide information to demonstrate phlorotannins are BZD agonists (positive allosteric modulators). This can be demonstrated using a functional assay based on electrophysiological measurements. Purified phlorotannin supplementation and dieckol potentiated the GABA-mediated inward current cultured neurons, and their activities were blocked by the co-application of a BZD antagonist flumazenil, such as the BZD agonist diazepam [18]. Flumazenil inhibits the sedative–hypnotic activity of diazepam by blocking the binding of diazepam to the BZD site of GABA_A_ receptors [89]. These results provide direct evidence that purified phlorotannins and dieckol act as positive allosteric modulators of GABA_A_ receptors. It has been demonstrated that eckstolonol is a partial BZD agonist based on electrophysiological measurements and pharmacophore modeling [4]. The GABAergic mechanism of phlorotannins has also been demonstrated through in vivo animal assays. The hypnotic activities of all phlorotannin preparations (except butanol and hexane fractions) and the individual constituents shown in Table 3 were completely blocked by the BZD antagonist flumazenil [4,17,18,72].

## 7. Conclusions

Numerous studies on the biological properties of phlorotannins and their constituents have been performed. Recently, marine polyphenol phlorotannins have been demonstrated to have sedative–hypnotic effects in animal models and clinical trials. In Korea, the MFDS has approved *E. cava* supplementation as a functional ingredient for health foods that help improve sleep quality. The sedative–hypnotic effects of phlorotannins suggest that they possess other neuropharmacological activities. It is necessary to demonstrate their anxiolytic or antidepressant effects and possible mechanisms of action. Red and green seaweeds contain non-phlorotannin polyphenol compounds. To date, the sedative–hypnotic effects of red or green seaweeds have not yet been reported. Therefore, these seaweeds could be promising raw materials for finding sedative–hypnotic compounds with novel structures or mechanisms.

## Figures and Tables

**Table 1:** In vivo evaluation methods for assessing hypnotic effects.

Methods	Pentobarbital-Induced Sleep Test	Polygraphic Recordings
Animal	ICR mice or SD rats	C57BL/6N mice or SD rats
Measurements	Righting reflex	EEG and EMG
Evaluation markers	Sleep latency, sleep duration, and sleep onset	Sleep latency, amount of NREMS and REMS, delta activity, sleep–wake episodes
Advantages	Short assay time, possible to screen many samples	Assessment of both sleep quantity and quality
Disadvantages	Impossible to evaluate sleep quality	Long assay time, high cost

Abbreviations: ICR, imprinting control region; SD, Sprague–Dawley; EEG, electroencephalogram; EMG, electromyogram; NREMS, non-rapid eye movement sleep; REMS, rapid eye movement sleep.

**Table 2:** Results from the pentobarbital-induced sleep test and polygraphic recordings studies on individual constituents of phlorotannins.

Compound	Methods (Dose) and Activities
Eckol	Pentobarbital-induced sleep test (50 mg/kg) duration ↑ [4]
Eckstolonol	Pentobarbital-induced sleep test (50 mg/kg) duration ↑ [4] Polygraphic recordings (50 mg/kg) NREMS ↑, latency ↓ Delta activity − [4]
Dieckol	Pentobarbital-induced sleep test (50 mg/kg) duration ↑ [4] Polygraphic recordings (150 mg/kg) NREMS ↑, latency ↓ Delta activity − [19]
Triphlorethol A	Pentobarbital-induced sleep test (50 mg/kg) duration ↑ [4] Polygraphic recordings (50 mg/kg) NREMS ↑, latency ↓ Delta activity − [83]
Fucodiphlorethol G	Pentobarbital-induced sleep test (50 mg/kg) duration ↑ [4]
6,6′-Bieckol	Pentobarbital-induced sleep test (50 mg/kg) duration ↑ [4]

NREMS, non-rapid eye movement sleep; −, not significant; ↑, increase; ↓, decrease.

**Table 3:** Results from in vitro GABAergic mechanism studies on preparations and individual constituents of phlorotannins.

Samples	Binding Affinity to the BZD Binding Site (IC_50_)	Functional Assay for the GABA_A_ Receptors
Preparations from *Ecklonia cava*		
Enzymatic extract	1.409 mg/mL [72]	-
Methanol extract	0.392 mg/mL [17]	-
Ethanol extract (EE)	0.127 mg/mL [17]	-
Ethyl acetate fraction from EE	0.019 mg/mL [17]	-
Butanol fraction from EE	0.103 mg/mL [17]	-
Hexane fraction from EE	0.141 mg/mL [17]	-
Purified phlorotannin supplement	0.012 mg/mL [4]	Positive allosteric activation to the GABA_A_ receptors [18]
Individual phlorotannin compounds		
Eckstolonol	2.422 μM [17]	Positive allosteric activation to the GABA_A_ receptors [4]
Eckol	1.739 μM [17]	-
Triphlorethol-A	7.180 μM [17]	-
Dieckol	4.991 μM [17]	Positive allosteric activation to the GABA_A_ receptors [18]

## Data Availability

Not applicable.

## References

[1] Lee S.H., Jeon Y.J. (2013). Anti-Diabetic Effects of Brown Algae Derived Phlorotannins, Marine Polyphenols through Diverse Mechanisms. Fitoterapia.

[2] Shibata T., Fujimoto K., Nagayama K., Yamaguchi K., Nakamura T. (2002). Inhibitory Activity of Brown Algal Phlorotannins against Hyaluronidase. Int. J. Food Sci. Technol..

[3] Chen L., Liu R., He X., Pei S., Li D. (2021). Effects of Brown Seaweed Polyphenols, a Class of Phlorotannins, on Metabolic Disorders: Via Regulation of Fat Function. Food Funct..

[4] Cho S., Yoon M., Pae A.N., Jin Y.H., Cho N.C., Takata Y., Urade Y., Kim S., Kim J.S., Yang H. (2014). Marine Polyphenol Phlorotannins Promote Non-Rapid Eye Movement Sleep in Mice via the Benzodiazepine Site of the GABAA Receptor. Psychopharmacology.

[5] Isaza Martínez J.H., Torres Castañeda H.G. (2013). Preparation and Chromatographic Analysis of Phlorotannins. J. Chromatogr. Sci..

[6] Khan F., Jeong G.J., Khan M.S.A., Tabassum N., Kim Y.M. (2022). Seaweed-Derived Phlorotannins: A Review of Multiple Biological Roles and Action Mechanisms. Mar. Drugs.

[7] Rajan D.K., Mohan K., Zhang S., Ganesan A.R. (2021). Dieckol: A Brown Algal Phlorotannin with Biological Potential. Biomed. Pharmacother..

[8] Kwon S., Yoon M., Lee J., Moon K.D., Kim D., Kim S.B., Cho S. (2019). A Standardized Phlorotannin Supplement Attenuates Caffeine-Induced Sleep Disruption in Mice. Nutrients.

[9] Wijesekara I., Yoon N.Y., Kim S.K. (2010). Phlorotannins from Ecklonia Cava (Phaeophyceae): Biological Activities and Potential Health Benefits. BioFactors.

[10] Lee S., Youn K., Kim D.H., Ahn M.R., Yoon E., Kim O.Y., Jun M. (2018). Anti-Neuroinflammatory Property of Phlorotannins from Ecklonia Cava on Aβ25-35-Induced Damage in PC12 Cells. Mar. Drugs.

[11] Kang M.C., Wijesinghe W.A.J.P., Lee S.H., Kang S.M., Ko S.C., Yang X., Kang N., Jeon B.T., Kim J., Lee D.H. (2013). Dieckol Isolated from Brown Seaweed Ecklonia Cava Attenuates Type II Diabetes in Db/Db Mouse Model. Food Chem. Toxicol..

[12] Li Y., Qian Z.J., Ryu B.M., Lee S.H., Kim M.M., Kim S.K. (2009). Chemical Components and Its Antioxidant Properties in Vitro: An Edible Marine Brown Alga, Ecklonia Cava. Bioorg. Med. Chem..

[13] Jang S.K., Yu J.M., Kim S.T., Kim G.H., Park D.W., Lee D.I., Joo S.S. (2015). An Aβ42 Uptake and Degradation via Rg3 Requires an Activation of Caveolin, Clathrin and Aβ-Degrading Enzymes in Microglia. Eur. J. Pharmacol..

[14] Wang C.H., Li X.F., Jin L.F., Zhao Y., Zhu G.J., Shen W.Z. (2019). Dieckol Inhibits Non-Small–Cell Lung Cancer Cell Proliferation and Migration by Regulating the PI3K/AKT Signaling Pathway. J. Biochem. Mol. Toxicol..

[15] Lee A.J., Ashkar A.A. (2018). The Dual Nature of Type I and Type II Interferons. Front. Immunol..

[16] Um M.Y., Lim D.W., Son H.J., Cho S., Lee C. (2018). Phlorotannin-Rich Fraction from Ishige Foliacea Brown Seaweed Prevents the Scopolamine-Induced Memory Impairment via Regulation of ERK-CREB-BDNF Pathway. J. Funct. Foods.

[17] Cho S., Yang H., Jeon Y.J., Lee C.J., Jin Y.H., Baek N.I., Kim D., Kang S.M., Yoon M., Yong H. (2012). Phlorotannins of the Edible Brown Seaweed Ecklonia Cava Kjellman Induce Sleep via Positive Allosteric Modulation of Gamma-Aminobutyric Acid Type A-Benzodiazepine Receptor: A Novel Neurological Activity of Seaweed Polyphenols. Food Chem..

[18] Kwon S., Jung J.H., Cho S., Moon K.D., Lee J. (2021). Dieckol Is a Natural Positive Allosteric Modulator of GABAA-Benzodiazepine Receptors and Enhances Inhibitory Synaptic Activity in Cultured Neurons. Nutr. Neurosci..

[19] Yoon M., Kim J.S., Seo S., Lee K., Um M.Y., Lee J., Jung J., Cho S. (2020). Dieckol, a Major Marine Polyphenol, Enhances Non-Rapid Eye Movement Sleep in Mice via the GABAA-Benzodiazepine Receptor. Front. Pharmacol..

[20] McCarthy B., O’Neill G., Abu-Ghannam N. (2022). Potential Psychoactive Effects of Microalgal Bioactive Compounds for the Case of Sleep and Mood Regulation: Opportunities and Challenges. Mar. Drugs.

[21] Hu Z., Oh S., Ha T.W., Hong J.T., Oh K.W. (2018). Sleep-Aids Derived from Natural Products. Biomol. Ther..

[22] Neubauer D.N. (2014). New and Emerging Pharmacotherapeutic Approaches for Insomnia. Int. Rev. Psychiatry.

[23] Roth T., Drake C. (2004). Evolution of Insomnia: Current Status and Future Direction. Sleep Med..

[24] Cho S., Shimizu M. (2015). Natural sleep aids and polyphenols as treatments for insomnia. Bioactive Nutraceuticals and Dietary Supplements in Neurological and Brain Disease.

[25] Fang X.S., Hao J.F., Zhou H.Y., Zhu L.X., Wang J.H., Song F.Q. (2010). Pharmacological studies on the sedative-hypnotic effect of Semen ziziphi spinosae (Suanzaoren) and Radix et Rhizoma Salviae miltiorrhizae (Danshen) extracts and the synergistic effect of their combinations. Phytomedicine.

[26] Yoon M., Kim J.S., Um M.Y., Yang H., Kim J., Kim Y.T., Lee C., Kim S.B., Kwon S., Cho S. (2017). Extraction Optimization for Phlorotannin Recovery from the Edible Brown Seaweed Ecklonia Cava. J. Aquat. Food Prod. Technol..

[27] Kadam S.U., Tiwari B.K., O’Donnell C.P. (2013). Application of Novel Extraction Technologies for Bioactives from Marine Algae. J. Agric. Food Chem..

[28] Cotas J., Leandro A., Monteiro P., Pacheco D., Figueirinha A., Goncąlves A.M.M., da Silva G.J., Pereira L. (2020). Seaweed Phenolics: From Extraction to Applications. Mar. Drugs.

[29] Shi J., Nawaz H., Pohorly J., Mittal G., Kakuda Y., Jiang Y. (2005). Extraction of Polyphenolics from Plant Material for Functional Foods—Engineering and Technology. Food Rev. Int..

[30] Sridhar A., Vaishampayan V., Senthil Kumar P., Ponnuchamy M., Kapoor A. (2022). Extraction Techniques in Food Industry: Insights into Process Parameters and Their Optimization. Food Chem. Toxicol..

[31] Bezerra M.A., Santelli R.E., Oliveira E.P., Villar L.S., Escaleira L.A. (2008). Response Surface Methodology (RSM) as a Tool for Optimization in Analytical Chemistry. Talanta.

[32] Huang Z., Bi R., Musil S., Pétursdóttir Á.H., Luo B., Zhao P., Tan X., Jia Y. (2022). Arsenic Species and Their Health Risks in Edible Seaweeds Collected along the Chinese Coastline. Sci. Total Environ..

[33] Šlejkovec Z., Kápolna E., Ipolyi I., van Elteren J.T. (2006). Arsenosugars and Other Arsenic Compounds in Littoral Zone Algae from the Adriatic Sea. Chemosphere.

[34] Hong Y.S., Song K.H., Chung J.Y. (2014). Health Effects of Chronic Arsenic Exposure. J. Prev. Med. Public Health.

[35] Mandal P. (2017). An Insight of Environmental Contamination of Arsenic on Animal Health. Emerg. Contam..

[36] Chen C.J., Wang S.L., Chiou J.M., Tseng C.H., Chiou H.Y., Hsueh Y.M., Chen S.Y., Wu M.M., Lai M.S. (2007). Arsenic and Diabetes and Hypertension in Human Populations: A Review. Toxicol. Appl. Pharmacol..

[37] Singh A.P., Goel R.K., Kaur T. (2011). Mechanisms Pertaining to Arsenic Toxicity. Toxicol. Int..

[38] Engel R.R., Hopenhayn-Rich C., Receveur O., Smith A.H. (1994). Vascular Effects of Chronic Arsenic Exposure: A Review. Epidemiol. Rev..

[39] Kim J., Yoon M., Yang H., Jo J., Han D., Jeon Y.J., Cho S. (2014). Enrichment and Purification of Marine Polyphenol Phlorotannins Using Macroporous Adsorption Resins. Food Chem..

[40] Pal P., Sen M., Manna A., Pal J., Pal P., Roy S., Roy P. (2009). Contamination of Groundwater by Arsenic: A Review of Occurrence, Causes, Impacts, Remedies and Membrane-Based Purification. J. Integr. Environ. Sci..

[41] Jiang Z., Wang Y. (2020). Stepwise Elution by High-Speed Counter-Current Chromatography Combined with a Modified Macroporous Resin to Isolate and Purify Antioxidant Phenolics from Discarded Jackfruit (*Artocarpusheterophyllus Lam*.) Peels. Anal Methods.

[42] Ren J., Zheng Y., Lin Z., Han X., Liao W. (2017). Macroporous Resin Purification and Characterization of Flavonoids from Platycladus Orientalis (L.) Franco and Their Effects on Macrophage Inflammatory Response. Food Funct..

[43] Wijesekara I., Kim S.K., Li Y., Li Y.X. (2011). Phlorotannins as Bioactive Agents from Brown Algae. Process Biochem..

[44] Joe M.-J., Kim S.-N., Choi H.-Y., Shin W.-S., Park G.-M., Kang D.-W., Kim Y.K. (2006). The Inhibitory Effects of Eckol and Dieckol from Ecklonia Stolonifera on the Expression of Matrix Metalloproteinase-1 in Human Dermal Fibroblasts. Biol. Pharm. Bull..

[45] Lee S.H., Kang S.M., Sok C.H., Hong J.T., Oh J.Y., Jeon Y.J. (2015). Cellular Activities and Docking Studies of Eckol Isolated from Ecklonia Cava (Laminariales, Phaeophyceae) as Potential Tyrosinase Inhibitor. Algae.

[46] Zhen A.X., Hyun Y.J., Piao M.J., Sameera Madushan Fernando P.D., Kang K.A., Ahn M.J., Yi J.M., Kang H.K., Koh Y.S., Lee N.H. (2019). Eckol Inhibits Particulate Matter 2.5-Induced Skin Keratinocyte Damage via MAPK Signaling Pathway. Mar. Drugs.

[47] Ha J.W., Song H., Hong S.S., Boo Y.C. (2019). Marine Alga Ecklonia Cava Extract and Dieckol Attenuate Prostaglandin E2 Production in HaCaT Keratinocytes Exposed to Airborne Particulate Matter. Antioxidants.

[48] Heo S.J., Ko S.C., Cha S.H., Kang D.H., Park H.S., Choi Y.U., Kim D., Jung W.K., Jeon Y.J. (2009). Effect of Phlorotannins Isolated from Ecklonia Cava on Melanogenesis and Their Protective Effect against Photo-Oxidative Stress Induced by UV-B Radiation. Toxicol. Vitro.

[49] Ko S.C., Cha S.H., Heo S.J., Lee S.H., Kang S.M., Jeon Y.J. (2011). Protective Effect of Ecklonia Cava on UVB-Induced Oxidative Stress: In Vitro and in Vivo Zebrafish Model. J. Appl. Phycol..

[50] Le Q.T., Li Y., Qian Z.J., Kim M.M., Kim S.K. (2009). Inhibitory Effects of Polyphenols Isolated from Marine Alga Ecklonia Cava on Histamine Release. Process Biochem..

[51] Lee J.W., Seok J.K., Boo Y.C. (2018). Ecklonia Cava Extract and Dieckol Attenuate Cellular Lipid Peroxidation in Keratinocytes Exposed to PM10. Evid.-Based Complement. Altern. Med..

[52] Zhang C., Li Y., Shi X., Kim S.-K. (2010). Inhibition of the expression on MMP-2, 9 and morphological changes via human fibrosarcoma cell line by 6,6′-bieckol from marine alga Ecklonia cava. BMB Rep..

[53] Yoon N.Y., Eom T.K., Kim M.M., Kim S.K. (2009). Inhibitory Effect of Phlorotannins Isolated from Ecklonia Cava on Mushroom Tyrosinase Activity and Melanin Formation in Mouse B16F10 Melanoma Cells. J. Agric. Food Chem..

[54] O’Sullivan A.M., O’Callaghan Y.C., O’Grady M.N., Queguineur B., Hanniffy D., Troy D.J., Kerry J.P., O’Brien N.M. (2011). In Vitro and Cellular Antioxidant Activities of Seaweed Extracts Prepared from Five Brown Seaweeds Harvested in Spring from the West Coast of Ireland. Food Chem..

[55] Quéguineur B., Goya L., Ramos S., Martín M.A., Mateos R., Guiry M.D., Bravo L. (2013). Effect of Phlorotannin-Rich Extracts of Ascophyllum Nodosum and Himanthalia Elongata (Phaeophyceae) on Cellular Oxidative Markers in Human HepG2 Cells. J. Appl. Phycol..

[56] Zhen A.X., Piao M.J., Hyun Y.J., Kang K.A., Fernando P.D.S.M., Cho S.J., Ahn M.J., Hyun J.W. (2019). Diphlorethohydroxycarmalol Attenuates Fine Particulate Matter-Induced Subcellular Skin Dysfunction. Mar. Drugs.

[57] Kang J., Kim S.C., Kim M.K., Boo H.J., Jeon Y.J., Koh Y.S., Yoo E.S., Kang S.M., Kang H.K. (2012). Effect of Dieckol, a Component of Ecklonia Cava, on the Promotion of Hair Growth. Int. J. Mol. Sci..

[58] Nagayama K., Shibata T., Fujimoto K., Honjo T., Nakamura T. (2003). Algicidal Effect of Phlorotannins from the Brown Alga Ecklonia Kurome on Red Tide Microalgae. Aquaculture.

[59] Kang M.C., Cha S.H., Wijesinghe W.A.J.P., Kang S.M., Lee S.H., Kim E.A., Song C.B., Jeon Y.J. (2013). Protective Effect of Marine Algae Phlorotannins against AAPH-Induced Oxidative Stress in Zebrafish Embryo. Food Chem..

[60] Choi H.S., Jeon H.J., Lee O.H., Lee B.Y. (2015). Dieckol, a Major Phlorotannin in Ecklonia Cava, Suppresses Lipid Accumulation in the Adipocytes of High-Fat Diet-Fed Zebrafish and Mice: Inhibition of Early Adipogenesis via Cell-Cycle Arrest and AMPKα Activation. Mol. Nutr. Food Res..

[61] Hwang H., Terada M., Shin H.-C. (2008). Single Dose Oral Toxicity and 4-Weeks Repeated Oral Toxicity Studies of Ecklonia Cava Extract. Seikatsu Eisei.

[62] Zaragozá M.C., López D., Sáiz M.P., Poquet M., Pérez J., Puig-Parellada P., Màrmol F., Simonetti P., Gardana C., Lerat Y. (2008). Toxicity and Antioxidant Activity in Vitro and in Vivo of Two Fucus Vesiculosus Extracts. J. Agric. Food Chem..

[63] Yang H., Yoon M., Kim J., Cho S. (2014). Acute Oral Toxicity of Phlorotannins in Beagle Dogs. Kor. J. Fish Aquat. Sci..

[64] Shin H.C., Kim S.H., Park Y., Lee B.H., Hwang H.J. (2012). Effects of 12-Week Oral Supplementation of Ecklonia Cava Polyphenols on Anthropometric and Blood Lipid Parameters in Overweight Korean Individuals: A Double-Blind Randomized Clinical Trial. Phytother. Res..

[65] Baldrick F.R., McFadden K., Ibars M., Sung C., Moffatt T., Megarry K., Thomas K., Mitchell P., Wallace J.M.W., Pourshahidi L.K. (2018). Impact of a (Poly)Phenol-Rich Extract from the Brown Algae Ascophyllum Nodosum on DNA Damage and Antioxidant Activity in an Overweight or Obese Population: A Randomized Controlled Trial. Am. J. Clin. Nutr..

[66] Paradis M.E., Couture P., Lamarche B. (2011). A Randomised Crossover Placebo-Controlled Trial Investigating the Effect of Brown Seaweed (Ascophyllum Nodosum and Fucus Vesiculosus) on Postchallenge Plasma Glucose and Insulin Levels in Men and Women. Appl. Physiol. Nutr. Metab..

[67] Um M.Y., Kim J.Y., Han J.K., Kim J., Yang H., Yoon M., Kim J., Kang S.W., Cho S. (2018). Phlorotannin Supplement Decreases Wake after Sleep Onset in Adults with Self-Reported Sleep Disturbance: A Randomized, Controlled, Double-Blind Clinical and Polysomnographic Study. Phytother. Res..

[68] Turck D., Bresson J., Burlingame B., Dean T., Fairweather-Tait S., Heinonen M., Hirsch-Ernst K.I., Mangelsdorf I., McArdle H.J., Naska A. (2017). Safety of Ecklonia Cava Phlorotannins as a Novel Food Pursuant to Regulation (EC) No 258/97. EFSA J..

[69] Catarino M.D., Silva A.M.S., Mateus N., Cardoso S.M. (2019). Optimization of Phlorotannins Extraction from Fucus Vesiculosus and Evaluation of Their Potential to Prevent Metabolic Disorders. Mar. Drugs.

[70] U.S. Food & Drug Administration (FDA) (2022). Recent Updates to the Notifications for New Dietary Ingredients. https://www.fda.gov/food/new-dietary-ingredients-ndi-notification-process/submitted-75-day-premarket-notifications-new-dietary-ingredients.

[71] Ministry of Food and Drug Safety (MFDS) (2022). Functional Ingredients for Health Functional Foods That Help Improve Sleep Quality. https://www.foodsafetykorea.go.kr/portal/healthyfoodlife/searchHomeHFDetail.do?prdlstReportLedgNo=2015021000031576.

[72] Cho S., Han D., Kim S.B., Yoon M., Yang H., Jin Y.H., Jo J., Yong H., Lee S.H., Jeon Y.J. (2012). Depressive Effects on the Central Nervous System and Underlying Mechanism of the Enzymatic Extract and Its Phlorotannin-Rich Fraction from Ecklonia Cava Edible Brown Seaweed. Biosci. Biotechnol. Biochem..

[73] Cho S., Yang H., Yoon M., Kim J., Kim D., Kim J., Kim S.B. (2014). Arousal Inhibitory Effect of Phlorotannins on Caffeine in Pentobarbital-Induced Mice. Fish Aquatic. Sci..

[74] Askari V.R., Rahimi V.B., Ghorbani A., Rakhshandeh H. (2016). Hypnotic Effect of Ocimum Basilicum on Pentobarbital-Induced Sleep in Mice. Iran Red. Crescent Med. J..

[75] Rakhshandeh H., Heidari A., Pourbagher-Shahri A.M., Rashidi R., Forouzanfar F. (2021). Hypnotic Effect of A. Absinthium Hydroalcoholic Extract in Pentobarbital-Treated Mice. Neurol. Res. Int..

[76] Rahimi V.B., Askari V.R., Tajani A.S., Hosseini A., Rakhshandeh H. (2018). Evaluation of the Sleep-Prolonging Effect of Lagenaria Vulgaris and Cucurbita Pepo Extracts on Pentobarbital-Induced Sleep and Possible Mechanisms of Action. Medicina.

[77] Cho S., Han D., Kim J., Yoon M., Yang H., Kim J. (2013). Potential claims and evaluation methods for sleep-promoting effects of foods. Food Sci. Ind..

[78] Yoon M., Kim J.S., Jo J., Han D., Cho S. (2014). Sleep-Promoting Effect of Ecklonia Cava: Ethanol Extract Promotes Non-Rapid Eye Movement Sleep in C57BL/6N Mice. Fish Aquatic. Sci..

[79] Masaki M., Aritake K., Tanaka H., Shoyama Y., Huang Z.L., Urade Y. (2012). Crocin Promotes Non-Rapid Eye Movement Sleep in Mice. Mol. Nutr. Food Res..

[80] Huang Z.L., Qu W.M., Eguchi N., Chen J.F., Schwarzschild M.A., Fredholm B.B., Urade Y., Hayaishi O. (2005). Adenosine A_2A_, but Not A1, Receptors Mediate the Arousal Effect of Caffeine. Nat. Neurosci..

[81] Revel F.G., Gottowik J., Gatti S., Wettstein J.G., Moreau J.L. (2009). Rodent Models of Insomnia: A Review of Experimental Procedures That Induce Sleep Disturbances. Neurosci. Biobehav. Rev..

[82] Fernando I.P.S., Lee W.W., Ahn G. (2022). Marine Algal Flavonoids and Phlorotannins; an Intriguing Frontier of Biofunctional Secondary Metabolites. Crit. Rev. Biotechnol..

[83] Yoon M., Cho S. (2018). Triphlorethol A, a Dietary Polyphenol from Seaweed, Decreases Sleep Latency and Increases Non-Rapid Eye Movement Sleep in Mice. Mar. Drugs.

[84] Lee S.M., Jeong H.H., Lee J.C., Park M.Y., Kim S.C. (2013). A Clinical Case Study on the Effects of Acupuncture Therapy and Ecklonia Cava Extract on Sleep Disturbances in ALS Patients. J. Acupunct. Res..

[85] Borja N.L., Daniel K.L. (2006). Ramelteon for the treatment of insomnia. Clin. Ther..

[86] Ebert B., Wafford K.A., Deacon S. (2006). Treating Insomnia: Current and Investigational Pharmacological Approaches. Pharmacol. Ther..

[87] Trevor A.J., Way W.L., Katzung B.G. (2007). Sedative-Hypnotic Drugs. Basic and Clinical Pharmacology.

[88] Erman M.K. (2005). Therapeutic options in the treatment of insomnia. J. Clin. Psychiatr..

[89] Brogden R.N., Goa K.L. (1991). Flumazenil. A Reappraisal of Its Pharmacological Properties and Therapeutic Efficacy as a Benzodiazepine Antagonist. Drugs.

